# Zinc Ointment

**Published:** 1853-03

**Authors:** 


*Zinc Ointment.*—Our attention has been directed to the following remarks of Dr. Copland, Part xvi. of his *Dictionary of Practical Medicine*, p. 935, on the subject of zinc ointment:—

"Great care should be taken in this affection, as well as in others for which ointments may be required, that they be recently made. In a case to which I was lately called, the zinc ointment was prescribed after the use of emollient applications, and was found quite rancid and most injurious at *five different Chemists* in the outskirts of the town where it was had; but when this ointment was procured from a respectable Chemist in town, it was quite successful."

To guard against a recurrence of this accident, we would suggest that where the demand is small, it would be better not to keep the ointment ready, but to mix it when wanted.—*Lond. Pharm. Jour. January*, 1853.

